# Hepatoprotective effect of essential oils of *Nepeta cataria L.* on acetaminophen-induced liver dysfunction

**DOI:** 10.1042/BSR20190697

**Published:** 2019-08-07

**Authors:** Juan Tan, Jing Li, Jilei Ma, Feng Qiao

**Affiliations:** 1 Department of Traditional Chinese Medicine, The Affiliated Hospital of Yan’an University, Shaanxi 71600, Yan’an, China; 2 Department of Traditional Chinese Medicine, Shaanxi People’s Hospital, Shaanxi Xi’an 710068, China; 3 Department of Clinical Laboratory, The First Affiliated Hospital of Zhengzhou University, Zhengzhou 450052, He’nan, China; 4 Department of Internal Medicine, Traditional Chinese Medicine Hospital of Yulin, Shaanxi Yulin 719000, China

**Keywords:** Essential oil, Hepatoprotective, Nepeta cataria L.

## Abstract

*Nepeta cataria L.* has long been used in folk food and medicine for several functions. Essential oils (EOs) were extracted from *Nepeta cataria L.* by supercritical fluid extraction. The results of animal experiments showed that EOs from *Nepeta cataria L.* significantly attenuated acetaminophen-induced liver damage. Further study confirmed that EOs were able to increase mRNA expression of uridine diphosphate glucuronosyltransferases (UGTs) and sulfotransferases (SULTs), as well as inhibit CYP2E1 activities, and thereby suppressed toxic intermediate formation. Nrf-2 activation might be involved in EOs-induced up-regulation of Phase II enzymes. Collectively, our data provide evidence that EOs protect the liver against acetaminophen-induced liver injury mainly by accelerating acetaminophen harmless metabolism, implying that EOs can be considered as a potential natural resource to develop hepatoprotective agent.

## Introduction

*Nepeta cataria L.* is a fragrant annual herb, widely distributed in Asia, Europe and North America since the Eastern Han Dynasty. It has been traditionally and popularly used as both food and medicine for thousands of years [1]. Since ancient times, the people in China have added the leaf of *Nepeta cataria L.* to noodles and other traditional Chinese foods for anti-microbial, anti-inflammation, cough-relieving, antioxidant and anti-cancer its abilities [2–5]. As a medicine, some clinical therapies are also reported in the literature. The search for other active compounds has led to the discovery and isolation of many phytochemicals and essential oils (EOs) with interesting anti-bacterial activity.

Drug-induced liver injury has become a major topic in the field of Hepatology and Gastroenterology. APAP overdose is the leading cause of drug-induced acute liver failure. Oxidative stress is considered to be the primary cellular event in acetaminophen-induced liver injury [6,7]. When taken at overdose, most of APAP is metabolized by Phase II conjugating enzymes, mainly sulfotransferase (SULT) and UDP-glucuronosyltransferase (UGT), converting it to nontoxic compounds which are then excreted with the urine. Of the remaining APAP, approximately 5–9% is metabolized by the cytochrome P450 enzymes (CYPs), mainly CYP2E1 into the highly reactive intermediate metabolite N-actyl-p-benzoquinone imine (NAPQI). Usually, NAPQI is rapidly detoxified by conjugating with glutathione (GSH). However, when Phase II metabolizing enzymes are saturated after APAP overdose, excessive NAPQ1 deplete GSH, leading to covalent binding of sulfhydryl groups in cellular proteins, this results liver oxidative stress [8,9].

In the present study, we found that EOs from *Nepeta cataria L.* have a liver protective ability on acetaminophen-induced liver injury.

## Materials and methods

### Supercritical fluid extraction of essential oil from *Nepeta cataria L.*

The EO from leaf of *Nepeta cataria L.* was extracted by supercritical CO_2_ extraction system. The settlement of parameters was selected on the basis of, temperature, pressure, static time, which had crucial efficiency on the EO extraction of supercritical fluid extraction (SFE). The primary parameters for EO extraction by SFE were selected according to practical experience. Supercritical CO_2_ was used as the extraction agent. The extraction procedure was operated under the optimized conditions: the extraction pressure was 35 MPa, extraction temperature was 50°C, flow rate of CO_2_ was 20 l/h, separation pressure was 6 MPa, separation temperature was 40°C and 1.5 h for the variable levels, respectively. The SFE coupled with the 5000-ml extraction vessel was used for EO from 5.0 kg powder of *Nepeta cataria L.* under the selected conditions. About 165 g of EO was collected.

### Experimental animals

The experimental protocol was reviewed and approved by the Ethics Committee of Fourth Military Medical University for the use of laboratory animals. A total of 50 (half male and half female) inbred Kunming mice (18–20 g) aged 4 weeks was obtained from the animal center of Fourth Military Medical University (Shanxi, China). The animals were kept under controlled conditions at temperature 22 ± 2°C, humidity 70 ± 4% with 12-h light–dark cycling.

### Animal treatments

Mice were randomly divided into five groups, ten mice were in each group (*n*=10, five males and five females). Control group: 1% Tween-80 aqueous solution (100 ml/kg); APAP group: 300 mg/kg; EOs-APAP group: EO was emulsified in a 1% Tween-80 aqueous solution, and then 100 ml/kg 1% Tween-80 were oral administration after intraperitoneal injection APAP (300 mg/kg) treatment 1 h; N-acetylcysteine (NAC)-APAP group: NAC (100 mg/kg) emulsified in a 1% Tween-80 aqueous solution and then it were oral administration after intraperitoneal injection APAP treatment 1 h; EOs group: EO was emulsified in a 95% Tween-80 aqueous solution (100 ml/kg). Mice were treated intragastrically with EOs after acetaminophen for 3 days.

### Determination of serum alanine aminotransferase and aspartate aminotransferase levels

Enzymatic activities of aspartate aminotransferase (AST) and alanine aminotransferase (ALT) in serum were evaluated by spectrophotometer using commercial diagnostic kits (Nanjing Jiancheng Institute of Biotechnology, Nanjing, China).

### Determination of hepatic reactive oxygen species, superoxide dismutase, catalase and malondiadehyde levels

Frozen liver tissues were homogenized in ice-cold PBS. The supernatants were collected after the homogenate was centrifuged at 3000 g, 4°C for 10 min. Superoxide dismutase (SOD), catalase (CAT) and malondiadehyde (MDA) levels by spectrophotometer using the commercially available assay kits as each manufacturer instructions (Nanjing Jiancheng Bioengineering Institute, Nanjing, China). The reactive oxygen species (ROS) levels were assay by fluorescence detector using commercial kits (Nanjing Jiancheng Bioengineering Institute, Nanjing, China). The protein concentration in tissue homogenates were measured by Bradford protein assay using bovine serum albumin as the standard (Nanjing Jiancheng Bioengineering Institute, Nanjing, China).

### Determination of related genes expression

Total mRNA was isolated from frozen liver tissues using a Total RNA kit (Tiangen, Beijing, China). Quantitative real-time PCR (qPCR) was carried out for the amplification of cDNA using 2×SYBR Green I PCR Master Mix (Vazyme, Nanjing, China). The PCR procedure consisted of 95°C for 30 s followed by 35 cycles of 95°C for 15 s, 58°C for 30 s and 72°C for 30 s. The PCR primers were used as shown in Table 1. The melting curve analysis was performed on the PCR products to verify primer specificity and product purity. A dissociation curve was performed for each plate to confirm the production of a single product. The relative abundance of each mRNA was calculated with the formula 2^−(ΔΔ*C*^_t_^)^, where ΔΔ*C*_t_ = (*C*_t__Target_ – *C*_t__GAPDH_) treatment − (*C*_t__Target_ − *C*_t__GAPDH_) control.

**Table 1:** Primers used for qPCR

Target gene	Sense 5′–3′	Antisense 5′–3′
Nrf2	GCTGATGGAGTACCCTGAGGCTAT	ATGTCCGCAATGGAGGAGAAGTCT
HO-1	TGCCAGTGCCACCAAGTTCAAG	TGTTGAGCAGGAACGCAGTCTTG
NQO1	GGAGACAGCCTCTTACTTGCCAAG	CCAGCCGTCAGCTATTGTGGATAC
GCLC	TGAGATTTAAGC CCCCTCCT	TTGGGATCAGTCCAGGAAAC
GSTA2	TCAGTAACCTGCCCACAGTGAAG	GCATGTTCTTGACCTCTATGGCTGG
UGT1A1	CACGCTGGGAGGCTGTTAGT	CACAGTGGGCACAGTCAGGTA
UGT1A6	CACGTGCTACCTAGAGGCACAG	GACCACCAGCAGCTTGTCAC
UGT1A9	GAAGAACATGCATTTTGCTCCT	CTGGGCTAAAGAGGTCTGTCATAGTC
SULT1A1	CCCGTCTATGCCCGGATAC	GGGCTGGTGTCTCTTTCAGAGT
SULT2A1	TAGGGAAAAATTTAGGGCCAGAT	TTGTTTTCTTTCATGGCTTGGA
CYP2E1	CACCGTTGCCTTGCTTGTCTG	CTCATGAGCTCCAGACACTTC
GAPDH	ACATGGCCTCCAAGGAGTAAGA	GATCGAGT TGGGGCTGTGACT

Abbreviations: GCLC, glutamate-cysteine ligase catalytic subunit; GSTA2, glutathione S-transferase A 2; HO-1, heme oxygenase-1; Nrf2, nuclear factor erythroid 2-related factor 2; NQO1, NAD(P)H: quinone oxidoreductase 1.

### Determination of pathological changes of liver

Taken a small piece of liver tissue and fixed it in 10% paraformaldehyde. After washing with distilled water, it is subjected to a series of operations such as dehydration, transparent, paraffin embedding and sectioning. The sections were counterstained with Hematoxylin after diaminobenzidine staining. Photomicrographs were taken with a digital camera.

### Determination of related protein expression

For nuclear factor erythroid 2-related factor 2 (Nrf2) expression analysis, the extraction and isolation of cytoplasmic and nuclear protein were performed using a Cytoplasmic and Nuclear Protein Extraction Kit (Beyotime, Nanjing, China) according to the manufacturer’s instructions. For CYP2E1 expression analysis, the extraction and isolation of microsomal protein were carried out as described previously [10]. The concentration of protein was determined by BCA assay kit (Beyotime, Nanjing, China). Equal amounts of protein extracts were subjected to SDS/polyacrylamide gel electrophoresis under reducing conditions on concentrate protein gel 5% (pH = 6.8) and separating protein gel 12% (pH = 8.8). The separated proteins were transferred to PVDF membranes using a tank transfer for 2 h at 200 mA in Tris-glycine buffer with 15% methanol. Membranes were blocked with 5% skimmed milk for 3 h and incubated for 12 h with anti-CYP2E1 (1:1500, BOSTER, Wuhan, China), anti-Nrf-2 (1:500, Bioss, Beijing, China), anti-GAPDH (1:1000, BOSTER, Wuhan, China) and anti-Lamin B (1:500, Bioss, Beijing, China) for 2 h at 37 °C. The secondary antibodies (IgG/HRP) were incubated for 2 h at 37°C. The images of the blots were visualized by ECL (Genshare, Xi’an, China).

### Statistical analysis

The results were presented as means of at least five measurements, duplicated for each set, having a coefficient of variation less than 5%. One-way ANOVA followed by Duncan’s multiple range test (*P*<0.05) with SPSS 20.0 (SPSS, Inc., Chicago, IL, U.S.A.).

## Results

### Effect of EOs on APAP-induced hepatotoxicity

**Figure 1:**
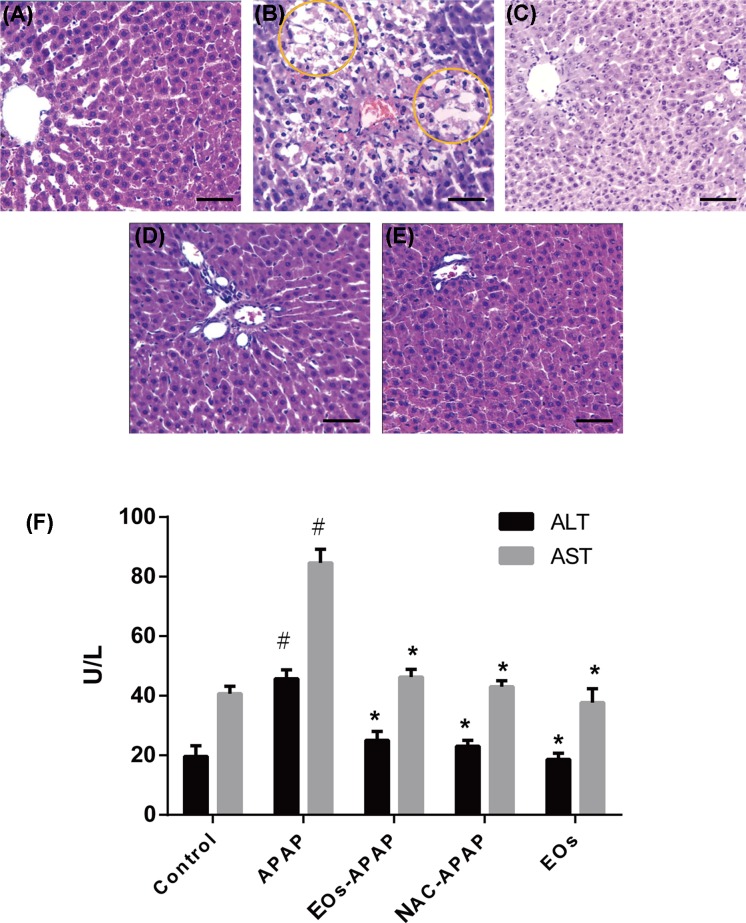
Serum markers of liver toxicity and liver changes in mice Notes: (**A–G**) The liver changes in mice. (**A**) Control group; (**B**) APAP treatment group; (**C**) APAP-EOs treatment group; (**D**) APAP-NAC treatment group; (**E**) EOs treatment group; bars = 20 μm. (**F**) The activity of ALT and AST. ^#^Significant compared with control group alone, *P*<0.05. ^*^Significant compared with model group alone, *P*<0.05.

APAP can induce liver injury by oxidative stress and inflammation [11,12]. ALT and AST were important markers of liver injury [13]. ALT and AST levels were significantly increased by 2.67-fold and 2.06-fold in APAP group, respectively, as compared with the control group (Figure 1F), suggestion APAP treatment induced liver injury. The levels of ALT and AST have no significant difference in EOs-APAP and NAC-APAP treatment group, suggestion EOs can prevent APAP-induced liver injury.

Pathological section results shown that control mice had normal hepatic architecture, APAP treatment mice exhibitions hepatocellular injury. More than half of the centrilobular hepatocytes were swollen with marked cytoplasmic vacuolation and condensed nuclei. Treatment with EOs from *Nepeta cataria L.* markedly attenuated the APAP-induced necrotic lesions (Figure 1A–E). Combine the results of Figure 1, EOs from *Nepeta cataria L.* have protective ability to prevent APAP-induced liver injury.

### Effect of EOs on hepatic antioxidant characters

**Figure 2:**
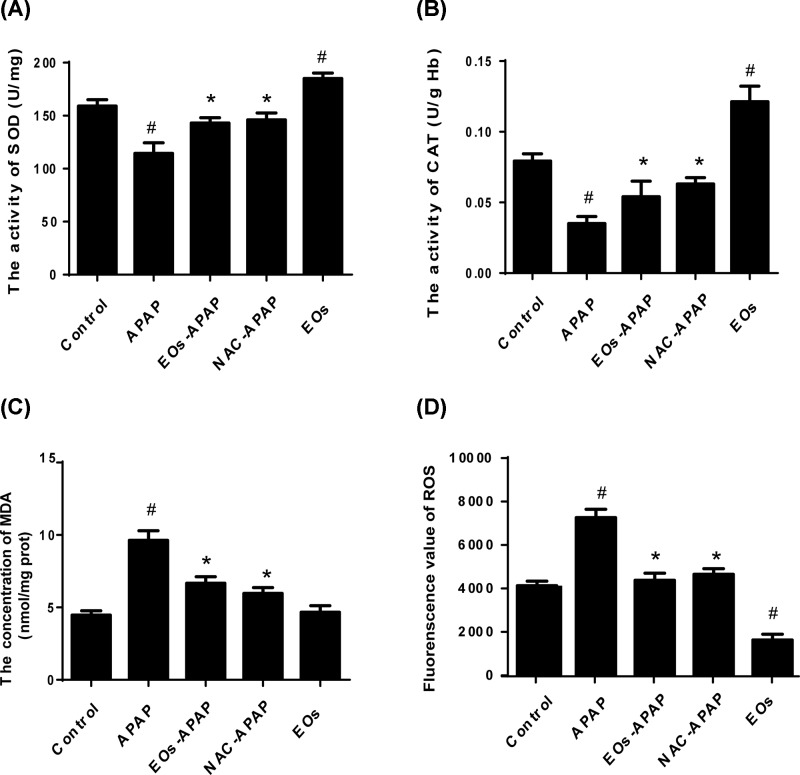
Effects of EOs on the oxidative stress markers in liver homogenate (**A**) The activity of SOD. (**B**) The activity of CAT. (**C**) The concentration of MDA. (**D**) Fluorescence value of ROS. ^#^Significant compared with control group alone, *P*<0.05. ^*^Significant compared with model group alone, *P*<0.05.

To investigate the effect of EOs on hepatic antioxidant characters, the levels of oxidative stress markers were examined in the model of APAP-induced mice. As shown in Figure 2, APAP treatment significantly increased the content of MDA and ROS by 2.38-fold and 1.92-fold, respectively. And APAP administration resulted in a significantly decreased the activity of SOD and CAT by 0.72-fold and 0.44-fold. Overdose APAP was metabolized by CYP2E1 enzymes into NAPQI, which undergoes chemical and enzymatic conjugation to GSH in APAP toxicity. This could lead to lipid peroxidation, antioxidant enzyme activities could be reduced and the levels of ROS were increased. Here, APAP increased MDA and ROS levels and decreased the activities of SOD and CAT, suggestions APAP-induced hepatic dysfunction is caused by oxidative stress.

EO-APAP administration prevented the decrease in SOD and CAT by 1.32-fold and 2.2-fold, and reduced the levels of ROS and MDA by 0.53-fold and 0.57-fold, respectively. EOs treatment not significantly increased the activities of SOD and CAT but significantly decreased the levels of ROS compared with the control group (*P*<0.05), suggestion EOs have the ability of ROS elimination.

### Effect of EOs on oxidative stress-related gene expression

**Figure 3:**
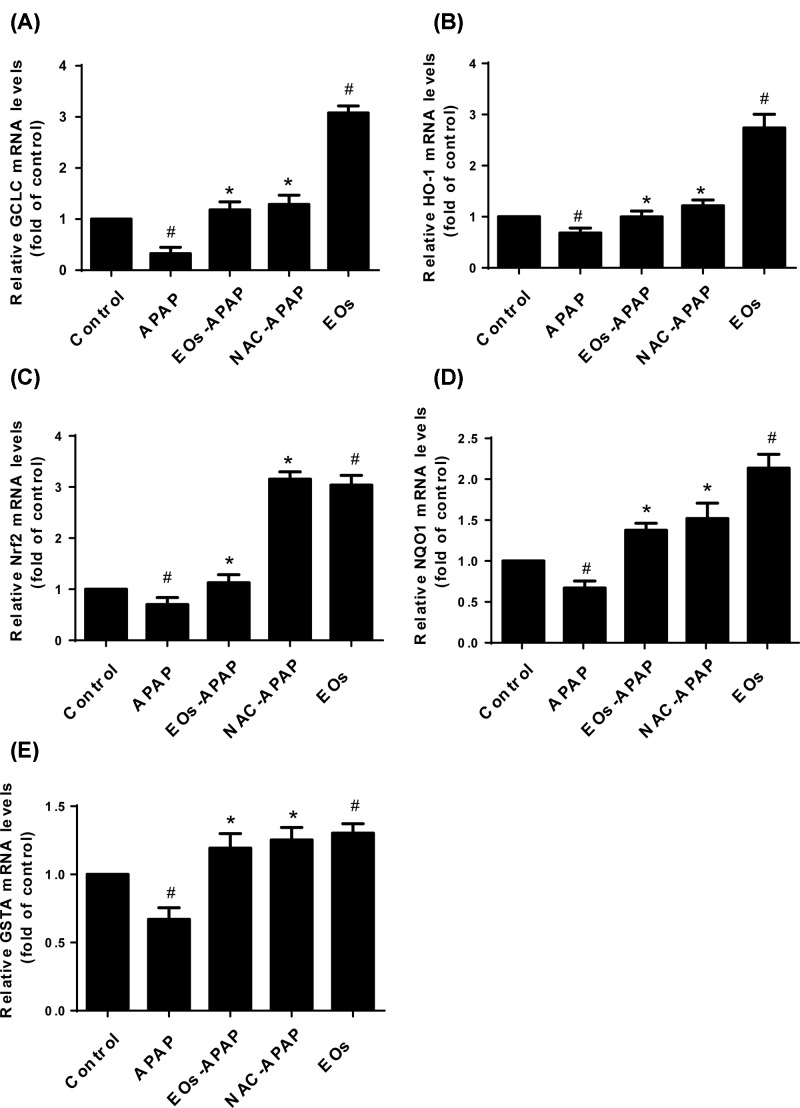
The levels of mRNA expression of oxidative stress-related gene ^#^Significant compared with control group alone, *P*<0.05. ^*^Significant compared with model group alone, *P*<0.05.

Nrf2 is a kind of transcription factor that modulates endogenous antioxidants and antioxidant enzymes [14–16]. ROS stimulates Nrf2 activation, which binds to the antioxidant response element and further activates transcription of gene encoding for antioxidants and detoxifications like heme oxygenase-1 (HO-1), NAD(P)H: quinone oxidoreductase 1 (NQO1), and glutathione-synthesizing enzymes, like glutamate-cysteine ligase catalytic subunit (GCLC) [17]. To investigate the effect of EOs on Nrf2, HO-1, NQO1, GCLC and glutathione S-transferase A 2 (GSTA2) gene expression, the mRNA of them was assayed by qPCR. As shown in Figure 3, APAP treatments were significantly decreased the levels of GCLC and GSTA, EOs-APAP treatment alleviate APAP induced decreased levels of GCLC and GSTA. EOs significantly increased the levels of Nrf2, HO-1, NQO1, GCLC and GSTA2, suggestion EOs can mediate antioxidant biological activities.

### Effect of EOs on APAP metabolism

**Figure 4:**
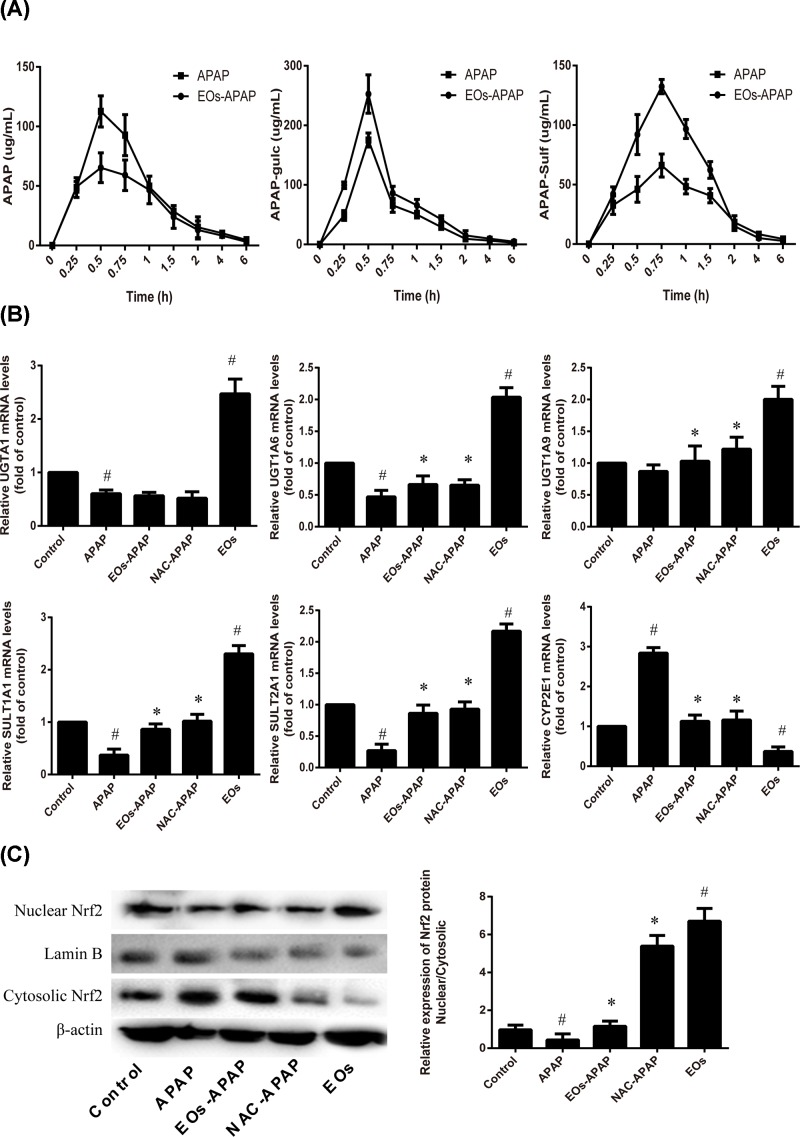
Effects of EOs on the APAP metabolic disposition (**A**) EOs can increase the APAP metabolized into APAP-gluc and APAP-sulf. (**B**) The mRNA expression of major enzymes involved in APAP lucuronidation. (**C**) The expression of Nuclear Nrf2 and Cyosolic Nrf2 was detected by Western blot. ^#^Significant compared with control group alone, *P<0.05*. ^*^Significant compared with model group alone, *P*<0.05.

APAP and its major conjugates in plasma were analyzed. As shown in Figure 4A, compared with APAP treatment group, the concentration of APAP was decreased, while APAP-gulc and APAP-sulf were increased in APAP-EOs treatment. The results show that EOs can increase the APAP metabolized into APAP-gluc and APAP-sulf.

APAP has two metabolism pathway in liver, one is non-toxicity pathway, APAP was metabolized into APAP-gluc and APAP-sulf by UGT family and SULT family and excreted in the blood and bile [18]. UGT1A6 and UGT1A1 are major enzymes involved in APAP glucuronidation. The increased levels of UGT1A6 and UGT1A1 enhanced the resistance to APAP toxicity [19]. In the present study, the levels of UGT1A1, UGT1A6, UGT1A9, SULT1A1, SULT2A1 were significantly increased after EOs treatments, suggestion EOs can enhance APAP metabolism by non-toxicity pathway. UGT family and SULT family are essential for inducing gene expression by Nrf2 with the consensus of TGAG/CNNNGC (N represents any base) [20]. As the result shows that EOs significantly increased the mRNA expression of Nrf2 (Figure 3C). The activated Nrf2 was transfer from cytoplasm into nuclear, the nuclear/cytosolic relative expression was significantly increased in EOs treatment group, suggestion EOs were induced Nrf2 transfer from cytoplasm to nucleus, then it leads to transcriptional activation of antioxidant enzymes, such as HO-1, SOD, CAT (Figures 2 and 3) and Phase II metabolic enzyme, such as UGT, SULT (Figure 4).

The second metabolism pathway of APAP was toxicity. Overdose APAP was metabolism to NAPQI by CYP2E1. In the present study, the expression of CYP2E1 was significantly increased by APAP treatment and decreased by EOs treatment (Figure 4). CYP2E1 deficient mice were resistant to the liver injury induced APAP, while the transgenic mouse expression human CYP2E1 were susceptible the conversion of APAP to NAPQI. Our results show that EOs inhibit CYP2E1 expression to against the liver injury.

Together, EOs induced the Nrf2 activity, the activated Nrf2 transcriptional activation of UGT and SULT. EOs were acceleration non-toxicity metabolism of APAP into APAP-gluc and APAP-sulf by enhance the levels of UGT and SULT, as well as inhibition of CYP2E1, which decreased the formation of NAPQI.

## Discussion

APAP is metabolized by sulfation, glucuronidation and CYP oxidation. The sulfation and glucuronidation pathways are considered detoxification routes, while the CYP oxidation pathway generates NAPQI, a reactive and toxic species. In case of acetaminophen toxicity, the Phase II conjugation enzymes are saturated, and a higher fraction is converted to NAPQI that leads to oxidative stress of liver. ROS is one of major factors in oxidative stress progress. Nrf2, which is likely activated by redox status changes induced by NAPQI. Mechanistically, the activated Nrf2 transfers from cytoplasm to nucleus, then it leads to transcriptional activation of antioxidant enzymes, such as NADPH: quinone oxidoreductase 1 (NQO1), HO-1, glutamate cysteine ligase (GCL) and GSTA, which increased the expression of SOD and CAT [21]. Besides, APAP caused severe liver injury characterized by significantly increased serum AST and ALT levels, ROS and hepatic MDA, as well as liver SOD, CAT depletions.

NAC can be used to detoxify for acetaminophen poisoning. It has the functions of anti-oxidation, scavenging oxygen free radicals, preventing DNA damage, regulating cell metabolic activity, regulating gene expression and signal transduction, and inhibiting the production of inflammatory mediators in the body [22]. NAC has the FDA approval for the treatment of potentially hepatotoxic doses of APAP. Therefore, we used NAC as the positive control drugs.

The first record of *Nepeta cataria L.* was documented in the ancient Chinese herbal book Ben-Cao-Gang-Mu (Ming Dynasty, by Shizhen Li). The book stated that the fired leaves and tender stems of *Nepeta cataria L.* could be served as food and tea. EO was extracted by different methods. SFE method was chosen in food industries, and has been extensively used to remove metal ions from various solid and liquid matrices of environmental samples. Different areas culturing *Nepeta cataria L.* have a variety of different ingredients, and uses for different functions. However, the function of *Nepeta cataria L.* was unclear.

In our studies, we found that EOs from *Nepeta cataria L.* up-regulated the expression and activities of Nrf2 and detoxification enzymes including UGTs and SULTs, as well as inhibited the activity of CYP2E1, which decreased plasma concentration of APAP and accelerated APAP harmless metabolism. Our data also showed that the effectiveness of EOs was as good as the NAC.

In summary, EOs from *Nepeta cataria L.* are effective in APAP-induced liver injury. They can be considered as a potential natural resource to develop hepatoprotective agent and feed additive instead of antibiotics. The results from the present study might supply useful functional food and better the understanding of the antioxidative characteristics of EOs from traditional herbal *Nepeta cataria L.*

## Abbreviations

ALT: alanine aminotransferase
AST: aspartate aminotransferase
CAT: catalase
CYP: cytochrome P450 enzyme
EO: essential oil
GCLC: glutamate-cysteine ligase catalytic subunit
GSH: glutathione
GSTA2: glutathione S-transferase A 2
HO-1: heme oxygenase-1
MDA: malondiadehyde
NAC: N-acetylcysteine
NAPQI: N-actyl-p-benzoquinone imine
Nrf2: nuclear factor erythroid 2-related factor 2
qPCR: quantitative real-time PCR
ROS: reactive oxygen species
SFE: supercritical fluid extraction
SOD: superoxide dismutase
SULT: sulfotransferase
UGT: uridine diphosphate glucuronosyltransferase
